# Neuropsychiatric symptoms as first manifestation of olfactory groove meningioma - importance of neuroimaging evaluation

**DOI:** 10.1192/j.eurpsy.2021.1095

**Published:** 2021-08-13

**Authors:** I. Vaz, D. Maia

**Affiliations:** Psiquiatria, Centro Hospitalar de Trás-os-Montes e Alto Douro, Vila Real, Portugal

**Keywords:** Neuropsychiatric, Neuroimaging, meningioma

## Abstract

**Introduction:**

Neuropsychiatric symptoms may be the first and only manifestation of brain tumours, while classic neurological symptoms and signs may be minimal or absent at first. These patients will often receive psychiatric treatments for prolonged periods before correct diagnosis.

**Objectives:**

To report the case of a patient with olfactory groove meningioma presenting with neuropsychiatric symptoms as a basis for discussion.

**Methods:**

Retrospective review of clinical notes, neuroimaging results and house photos. Literature review.

**Results:**

A 66-year-old woman was brought by police to the psychiatric emergency department Her neighbours had notified authorities of a bad smell, and police found the house was loaded with garbage. The patients reported depressive symptoms in the last 6 months, including apathy, anhedonia, social isolation, decreased appetite and insomnia; loss of basic skills such as cooking or cleaning; she also reported dizziness and two episodes of urinary and faecal incontinence in public. The patient had a history of being medicated for depression between 2000 and 2006. Currently she was taking only alprazolam 1 mg daily. During evaluation she was conscious, oriented and cooperative, with evident hypomimia, psychomotor inhibition and indifferent attitude. Cranial nerve function was preserved except for anosmia. Cranial CT and MRI showed a solid extra-axial tumour of 5.2x3.5x4.9 centimetres compatible with meningioma of the olfactory groove, and she was referred to Neurosurgery for surgical intervention.

**Figure:**
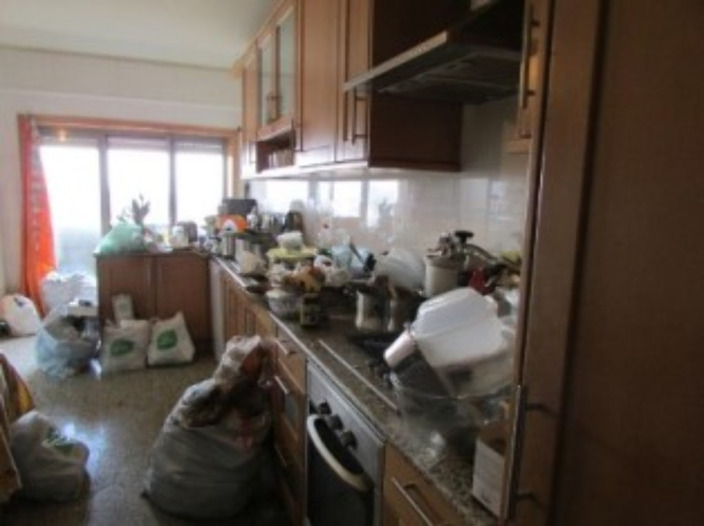

**Figure:**
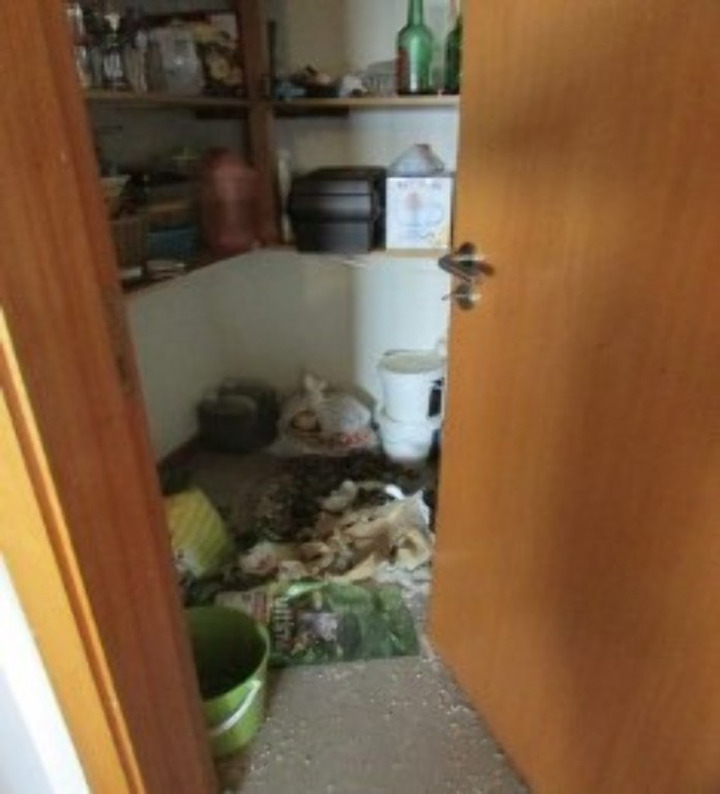

**Figure:**
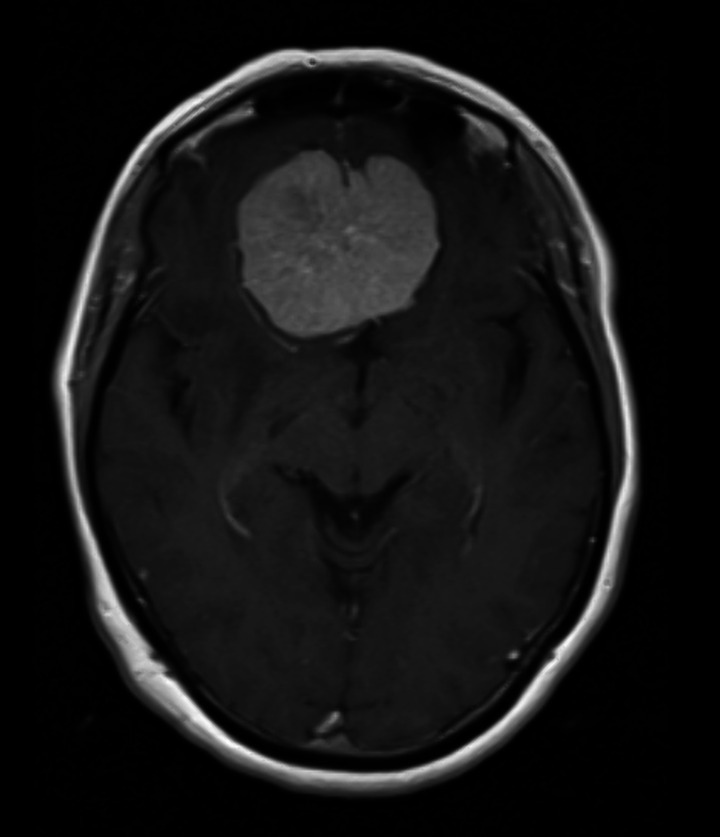

**Conclusions:**

This case illustrates the importance of a thorough organic evaluation, including neuroimaging, in the differential diagnosis of patients with atypical symptoms before making a psychiatric diagnosis and instituting treatment.

